# Eyedrop Vaccination Induced Systemic and Mucosal Immunity against Influenza Virus in Ferrets

**DOI:** 10.1371/journal.pone.0157634

**Published:** 2016-06-22

**Authors:** Sangchul Yoon, Eun-Do Kim, Min-Suk Song, Soo Jung Han, Tae Kwann Park, Kyoung Sub Choi, Young-Ki Choi, Kyoung Yul Seo

**Affiliations:** 1 Department of Ophthalmology, Severance Hospital, Institute of Vision Research, Yonsei University College of Medicine, Seoul, 03722, Republic of Korea; 2 Brain Korea 21 Project for Medical Science, Yonsei University, Seoul, 03722, Republic of Korea; 3 Department of Ophthalmology, Soonchunhyang University College of Medicine, Soonchunhyang University Bucheon Hospital, Gyeonggi-do, 14584, Republic of Korea; 4 College of Medicine and Medical Research Institute, Chungbuk National University, Cheongju, 28644, Republic of Korea; 5 Department of Ophthalmology, National Health Insurance Corporation Ilsan Hospital, Gyounggi-do, 10444, Republic of Korea; 6 Department of Ophthalmology, National Medical Center, Seoul, 04564, Republic of Korea; University of South Dakota, UNITED STATES

## Abstract

We investigated eyedrop vaccination (EDV) in pre-clinical development for immunological protection against influenza and for potential side effects involving ocular inflammation and the central nervous system (CNS). Live attenuated influenza EDV, CA07 (H1N1), PZ-4 (H1N2) and Uruguay (H3N2), induced both systemic and mucosal virus-specific antibody responses in ferrets. In addition, EDV resulted in a clinically significant protection against viral challenge, and suppression of viral replication in nasal secretion and lung tissue. Regarding safety, we found that administered EDV flow through the tear duct to reach the base of nasal cavity, and thus do not contact the olfactory bulb. All analyses for potential adverse effects due to EDV, including histological and functional examinations, did not reveal significant side effects. On the basis of these findings, we propose that EDV as effective, while being a safe administration route with minimum local side effects, CNS invasion, or visual function disturbance.

## Importance

Ferrets are superior animal models to mice for modified virus-based vaccine experiments because they are susceptible to a wide range of human influenza strains. Additionally, they are ideal for studying the efficacy of eyedrop vaccination (EDV) as their visual system largely similar to that of humans. In this study, we demonstrate that EDV can induce satisfactory immunity and protection against influenza in ferrets and confirmed the lack of any potential contraindications involving the central nervous or ocular systems through histologic and functional examination. This study accomplishes an important step forward to the human application of EDV in humans by establishing their safety and effectiveness for influenza vaccine immunization.

## Introduction

Mucosal vaccines have considerable advantages over those delivered through parenteral routes, since they can induce both systemic and mucosal antigen-specific immune responses and can be easily administered [1]. These immunizations are often administered via oral or intranasal routes, as exemplified with the oral polio vaccine (OPV) and recently approved FluMist^®^ influenza vaccine, respectively [2]. Compared to injectable vaccines that only elicit a systemic immune response, those given through these routes are also capable of inducing mucosal immune reactions associated with intestinal IgA production that enhances protective immunity and the prevention of human-to-human transmission [3]. While oral vaccinations are often ineffective due to frequent proteolytic degradation and inactivation during the gastrointestinal passage, intranasal vaccination has been studied in the context of respiratory infection and led to the commercialization of a licensed intranasal influenza vaccine [2–4]. Notably, intranasal spray is now considered as a new therapeutic approach for central nervous system (CNS) disorders because it can cross the blood-brain barrier [5–7]; however, this serve as a serious limitation for vaccination purposes due to the possibility of adverse neurological side effects.

The eye mucosa shares numerous immunologic features with its nasal counterpart, and has proven to be an effective antigen (Ag) delivery route by previous studies in fowls, bovine, goats, and chicken models of immunization [8–12]. Importantly, a recent investigation in mice determined that animals immunized with an influenza eyedrop vaccine (EDV) were protected from lethal pathogen infection [13]. Nevertheless, the clinical significance of these results is inherently compromised since mice are not natural hosts of influenza.

Ferret is one of the most appropriate animal models for the study of influenza EDV for several reasons. First, ferrets are widely used in the study of visual system because their ocular anatomy and physiology are similar to those of humans [14]. Second, ferrets have shown to be good model to investigate the pathogenesis and transmission of influenza since they exhibit a similar level of susceptibility and clinical response to human influenza virus in terms of clinical presentation and respiratory physiology [15–17]. Lastly, compared to the nonhuman primates, ferrets are highly preferable in terms of availability, cost of caring, and regulations associated with procurement and maintenance.

This study aims to evaluate the safety and efficacy of EDV in a ferret model of influenza. Previous investigations detailing immune provocation and acquired protection in mouse models are extended with our analyses in ferrets by providing critical commentary data on clinical presentation and organ histology. Furthermore, we have also been able to address potential safety concerns relating to adverse effects on the CNS and ocular inflammation.

## Materials and Methods

### Ethics statement

All experiments related to vaccination and virus infection of animal subjects along with the sample preparation of the ferrets were conducted in strict accordance and adherence to relevant Council of the Republic of Korea and international guidelines regarding animal handling approved by the Animal Use and Care by Laboratory Animal Research Centre (LARC; permit #BLS-ABSL-12-010) of Chungbuk National University (Cheong-ju, Korea), a member of the International Animal Care and Use Committee (IACUC).

All experiments using micro-CT or electroretinalgram to animal subjects were conducted in strict accordance and adherence to relevant national and international guidelines regarding animal handling approved by the Institutional Animal Care and Use Committee (IACUC) of Yonsei University Health System (Seoul, Korea; permit #2011–0137).

### Viruses

The live-attenuated viruses (Table 1) used in this study were adapted in egg at 27°C (cold adaptation, ca) more than 10 times. The inefficient growth of the ca, live-attenuated viruses compared to wild type corresponding viruses (10–100 fold decreases) was confirmed at 37°C. The human and animal infectious influenza viruses (Table 1) used for challenge in this study were provided by Chungbuk National University (Cheongju, South Korea) and amplified in 10-day-old embryonated chicken eggs. Viruses were serially diluted (10-fold) prior to infection in Mardin-Darby canine kidney cells and the 50% tissue culture infective doses (TCID_50_) was then calculated by the Reed-Muench method [18]. Stock viruses were kept at -82°C, and thawed right before use. Viral growth was determined by observing changes in cellular morphology (cytopathic effects) and hemagglutinin (HA) assay.

**Table 1:** List of influenza viruses used for vaccination and challenge in this study.

Group (subtype)	Virus strain	Abbreviation	Challenge dose (TCID_50_)	Similarity
H1N1				
Vaccination	A/California/7/09 x PR8^a^	CA07 (H1N1)	-	-
Challenge	A/California/04/09	CA04 (H1N1)	10^5^	Homologous
H1N2				
Vaccination	Sw/Korea/PZ4/06^b^	PZ-4 (H1N2)	-	-
Challenge	A/Sw/Korea/1204/09	Sw09 (H1N2)	10^6^	Heterologous
H3N2				
Vaccination	A/Uruguay/716/07x PR8^a^^,^^c^	Uruguay (H3N2)	-	-
Challenge	A/Hongkong/68	HK68 (H3N2)	10^6^	Heterologous

^a^ A/Puerto Rico/8/34; this virus is used as a backbone strain.

^*b*^ Cross-reactive HI titer between Sw/Korea/PZ4/06 and Sw/Korea/1204/09 is 2 fold lower than the HI titer of homologous Sw/Korea/PZ4/06 virus (160 Vs 320 HI units).

^*c*^ Cross-reactive HI titer between Uruguay/716/07xPR8 and Hongkong/68 is 0.

### Vaccination and virus challenge

15- to 16-week-old ferrets were purchased from Marshall BioResources (North Rose, NY, USA). All animals were confirmed seronegative for all vaccine strains of influenza A viruses used in this study, including CA07 (H1N1), PZ-4 (H1N2), and Uruguay (H3N2) by serologic assay. For conjunctival immunizations, ferrets were anesthetized by inhalation of isoflurane, and then 10^5 TCID_50_ of the CA07 (H1N1), PZ-4 (H1N2), and Uruguay (H3N2) live attenuated influenza vaccines (LAIVs) in 100 μL PBS were dropped to each eye (200μL/head) twice with a two week interval. Two weeks after the second immunization, ferrets were intranasally instilled with 1.0 mL volume of 10^5 TCID50/mL for the CA04 (H1N1) and 10^6 TCID_50_/mL for the Sw09 (H1N2) and HK68 (H3N2) for virus challenge [19]. Body temperatures and body weights of infected ferrets were monitored daily. There was no death after the viral infection.

### Experimental sampling

The ferrets were anesthetized with Zoletil 50^®^ (125mg zolazepam and 125mg tiletamine hydrochloride [Vibrac, Carros, France]; 0.2 mg/kg of body weight) and Rompun^®^ (2% xylazine hydrochloride [Bayer Animal Health, Leverkusen, Germany]; 5 mg/kg of body weight) administered intramuscularly 10 min before sampling. Serum samples from ferrets were collected for HI assays two weeks after each vaccination. Nasal secretion samples were collected in 1× PBS with antibiotics two weeks after every vaccination for HI assay or at 1, 3, 5, 7, and 9 days post infection (dpi) after viral challenge for titer assays. For the preparation of lung tissue samples, ferrets were euthanatized by CO_2_ inhalation. The lung tissues were collected at 5 dpi for histopathology or homogenized in 1× phosphate-buffered saline (PBS) containing antibiotics for virus titration. Tissue homogenates of both lobes were clarified by centrifugation at 12,000*g*, and supernatants were then transferred to fresh tubes. All samples were stored at -82°C until use.

### Serologic assays

Hemagglutination inhibition (HI) assays were performed as previously described to determine the seroprevalence of vaccinated and challenged viruses [20]. Briefly, serum samples were heat inactivated at 56°C for 30 min and pretreated with receptor-destroying enzyme (RDE) from *Vibrio cholerae* (Denka Seiken; Tokyo, Japan) to remove non-specific serum inhibitors. Sera were then analyzed for the presence of virus-specific antibody by HI assays with 0.5% turkey red blood cells (RBC). The HI titer was determined by the reciprocal of the last dilution that contained turkey RBCs with no agglutination. Neutralization tests performed on selected HI-positive sera to confirm results and used to determine MDCK cell infection and expressed as the reciprocal of the highest dilution of serum that gave 50% neutralization of 100 TCID_50_ of virus after incubation at 37°C for 72 h [21, 22]. Hemagglutination assays were performed according to WHO/World Organization for Animal Health (OIE) recommendations [23].

### Histology

The lungs or eye tissue of ferrets from each group were harvested at 5 dpi or 24 hour, respectively. Tissues were fixed in 10% neutral-buffered formalin, embedded in paraffin, sectioned (4 μm), and examined in the pathology laboratory of Chungbuk National University Hospital or Yonsei University College of Medicine. Histological assessment was performed by standard hematoxylin and eosin staining and light microscopy at 200× magnification. In blind fashion, either left or right lobes of five lungs were examined with five to seven slides per each lobe. Semi-quantitative analysis of lung inflammation severity in influenza virus-challenged ferrets was performed with some modification as reported elsewhere [24] for the alveoli. For the severity of inflammation in the alveoli, we scored (1–2) no infiltration of inflammatory cells and intact alveoli size, (3–4) mild infiltration of inflammatory cells and mildly shrunken alveoli, and (5–6) marked infiltration of inflammatory cells and extremely shrunken or disappeared alveoli. The cumulative scores for severity and size of inflammation provided the total score per animal.

### Micro-CT

Imaging was performed using a volumetric CT scanner (NFR-Polaris-G90MVC: NanoFocusRay, Iksan, Korea) as previously described with minor modifications [25]. Briefly, images were acquired at 65 kVp, 115 μA, 142-millisecond per frame, and with 700 views. The estimated radiation dose was ~150 mGy using this image acquisition protocol. Images were reconstructed using the volumetric cone-beam reconstruction (FDK) off line mode. The reconstruction image size was 1,204 × 1,024 pixels, and 512 slices were acquired. The final reconstructed data was converted to the Digital Imaging and Communications in Medicine (DICOM) format to make 3D-rendered imaging using 3D-rendering software (Lucion, MeviSYS, Seoul, Korea). For in vivo animal CT imaging, ferrets were anesthetized and imaged for a baseline prior to treatment with VISISENSE injection 320 contrast agent at a dose of 0.5 μL/g body weight (2.5 μmol Au/g body weight) via the ocular or intranasal administration. Ferrets were euthanatized by CO_2_ inhalation 30 min after the agent treatment. The brains of ocular or intranasal-treated ferrets were taken out in order to acquire CT mages.

### Electroretinalgram (ERG)

The ferrets were anesthetized with Zoletil 50^®^ (125mg zolazepam and 125mg tiletamine hydrochloride [Vibrac, Carros, France]; 0.2 mg/kg of body weight) and Rompun^®^ (2% xylazine hydrochloride [Bayer Animal Health, Leverkusen, Germany]; 5 mg/kg of body weight) administered intramuscularly 10 min before ERG examinations. Pupils were maximally dilated with achieved with 0.5% phenylephrine hydrochloride and 0.5% tropicamide (Mydrin-P^®^, Santen, Osaka, Japan). Animals were placed in a special holding system to prevent unfavorable movement during full-field electroretinogram (ERG) recording.

Full-field ERG recording was performed with RETIscan^®^ (Roland Consult, Wiesbaden, Germany) in the same examination room. A Goldring electrode^®^ with diameter of 3 mm (Roland Consult, Wiesbaden, Germany) was placed on the corneal surface as an active electrode using 0.3% hypromellos (GenTeal^®^, Novartis, Basel, Switzerland), while a second reference electrode was placed on the fornix of the same eye. Concentric subdermal needle electrodes (Roland Consult, Wiesbaden, Germany) were then used as a ground electrode after insertion into the tail of the animal.

Full-field ERG was recorded following the standards of International Society of Clinical Electrophysiology of Vision (ISCEV). Full-field stimulation was produced using Ganzfeld stimulator of the RETIscan unit (Roland Consult, Wiesbaden, Germany), which was positioned just in front of animal’s face. To assess rod responses by ERG, the animals were dark-adapted for 12 hours before anesthesia. A dim white flash with a stimulus strength of 0.01 cd·s/m^2^ was used for dark-adapted 0.01 ERGs with intervals of 2 seconds between flashes. A white 3.0 cd·s/m^2^ flashes were produced in dark-adapted eye for dark-adapted 3.0 ERGs and oscillatory potentials recordings. Subsequently, light-adapted ERGs recordings were performed after 15 min of light adaptation. Light-adapted 3.0 ERGs elicited by white flashes at an intensity of 3.0 cd·s/m^2^ under white background of 30 cd/m^2^. Furthermore, 30-Hz flicker responses were recorded using white light flashes at an intensity of 3.0 cd·s/m^2^ and a rate of 30 stimuli per second (30Hz). All of responses were amplified at 10,000 times, and were filtered with a band pass between 1 and 300Hz. Five waveforms for each response were averaged to reduce variability and background noise. ERG abnormality is reflected in alterations in A- and B-wave amplitude. A-wave amplitude measures the trough of the negative deflection as difference from the baseline value. B-wave amplitude measures the difference between the trough of A-wave and the peak of B-wave recording. Recordings of full-field ERG were performed for both eyes prior to the vaccination and 1 day after the treatment, respectively. Vaccination was applied only on right eyes, and left eyes were considered as a control eye.

For statistical analysis of the ERG parameters, the paired t-test was used to assess the difference between the values before and after the vaccination, respectively. The differences were considered to be significant when P-value was less than 0.05.

### Data and statistical analyses

Data were expressed as the mean ± SD, and statistical analyses were conducted by the student’s *t-test* (Microsoft Office Excel program). Number of animals in all groups was 3 or 4. For the data normality test, the Shapiro-Wilk test was used. All data was checked before we use the student’s t-test and concluded that all data came from a normal distribution.

## Results

### Induction of systemic and mucosal immune responses in ferrets by eyedrop Live-Attenuated Influenza Vaccine (LAIV)

**Fig 1:**
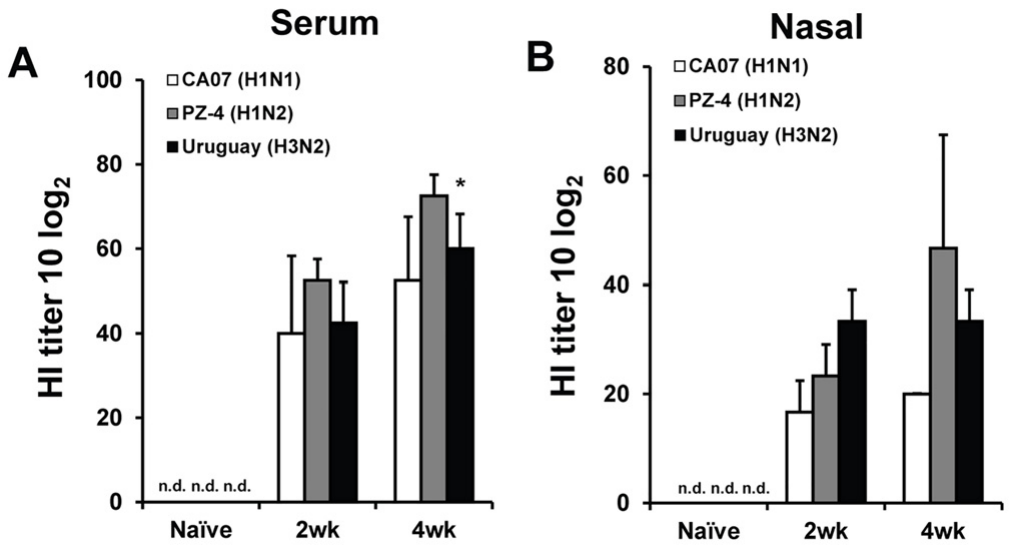
Eyedrop vaccination with live attenuated influenza vaccine (LAIV) elicits immunological responses in ferrets. Three groups of ferrets (n = 3) were administrated with two doses of CA07 (H1N1) or PZ-4 (H1N2) or Uruguay (H3N2) LAIV eyedrops, respectively. Levels of anti-LAIV HI titers were measured by HI assay in serum (A) and nasal lavage (B) samples, respectively, two weeks after the first (2wk) and the second (4wk) vaccination. *p < 0.05 compared with the findings in the 2 weeks group; n.d., non-detected. Statistical analyses were conducted by the student’s *t-test*.

To evaluate the capacity for the eyedrop influenza vaccine to elicit protective immunity in ferrets, we separately administered three different strains of LAIV (Table 1) to three groups of ferrets (n = 3 or 4) twice with two weeks interval. Serum and nasal lavage samples were collected after each immunization to assess anti-LAIV HI titer levels. As shown in Fig 1A, all samples from influenza-naïve ferrets were negative for virus-specific antibodies (Abs); however, serum HI titers were significantly increased in all immunized ferrets following the first vaccination as compared to those of the naïve group. CA07- and Uruguay-vaccinated groups exhibited a 40% and 50% increase in the serum HI levels after the second vaccination, respectively, when compared to the primary vaccination titers (Fig 1A). In agreement, the HI titer levels of nasal lavage samples isolated from each group following the first vaccination (at two weeks after the priming; 2wk) were significantly increased compared to those observed in naïve counterparts (Fig 1B). The average HI titer in nasal lavage samples was lower than those of serum samples across all immunized animals at 24.4 and 45 HI, respectively. No significant increases in nasal lavage HI titers were present between the first (2wk) and second (4wk) vaccinations. Moreover, we tried to measure the increase of IgG Ab by ELISA, and, unfortunately, we failed to detect any significant increase due to the high level of non-specific binding of secondary anti-ferret IgG or IgA Abs in PBS sample (S1 Fig). However, since nasal lavage and blood serum samples mainly contain secreted IgA and IgG, respectively, the observable increase in HI titers from nasal lavage samples suggest that an induction of LAIV-specific IgA Abs secretion occurred. Therefore, these results demonstrate that eyedrop LAIV vaccination is sufficient to provoke Ag-specific systemic and mucosal immune responses in ferrets.

### EDV protects ferrets from intranasal influenza virus challenge

**Fig 2:**
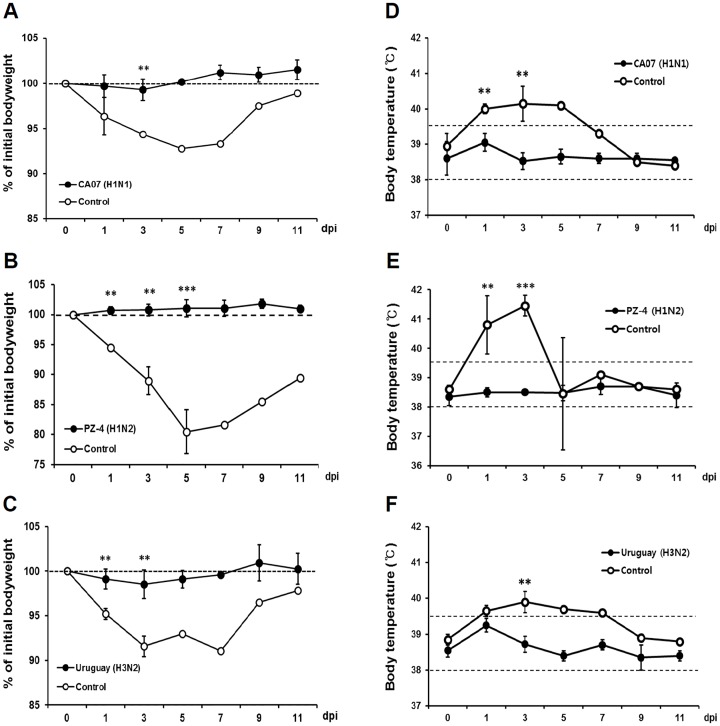
Eyedrop influenza vaccine protects ferrets from influenza virus challenge. At two weeks after the last eyedrop vaccination, ferrets were challenged i.n. with 1 × 10^5^ TCID50 of CA04 (H1N1) on CA07 (H1N1) LAIV-vaccinated group (A, D) or 1 × 10^6^ TCID50 of Sw09 (H1N2) or HK69 (H3N2) on PZ-4 (H1N2) LAIV-vaccinated (B and E) or Uruguay (H3N2) LAIV-vaccinated (C and F) group, respectively. Body weight (A-C) and body temperatures (D-F) were monitored for 11 days with an interval of two days. Dotted lines in figures D-F: normal body temperature range. **p < 0.01, ***p < 0.001 compared with the findings in the PBS control group (n = 3 for each group). Statistical analyses were conducted by the student’s *t-test*.

To evaluate whether LAIV administration could confer a protective immunity against influenza infection, four immunized ferrets in each EDV group or PBS treated ferrets as control group were challenged with CA04 (H1N1), Sw09 (H1N2), and HK68 (H3N2) administered intranasally. Changes in body weight and temperature were then monitored to assess disease onset (Fig 2). Mock (PBS)-immunized ferrets significantly began to lose about 10% of initial body weight compared to EDV vaccinated groups in CA04 (H1N1) and in HK69 (H3N2) infected ferrets or about 20% loss compared to EDV vaccinated groups in Sw09 (H1N2) infected ferrets (Fig 2A–2C). However, all ferrets of EDV groups showed no body weight loss. Notably, PZ-4 (H1N2)-vaccinated ferrets gained weight after Sw09 (H1N2) viral challenge, while the control group demonstrated significant weight loss until day 5 post-infection (Fig 2B).

Likewise, all ferrets in the PBS treated control groups showed significantly higher rise of temperature compared to their counterparts that spiked to over 40°C in three days following influenza virus challenge (normal body temperature ranges from 38.5 to 39.5°C). Additionally, animals infected with Sw09 (H1N2) virus exhibited the highest temperature in control ferrets, concordant with the increased weight loss in shown in Fig 2B (Fig 2E). In contrast, ferrets of all types of EDV groups showed no noticeable signs of fever throughout the monitoring period; although, their body temperature did rise ≤0.5°C after 24 h, but returned to normal ranges within 3 days (Fig 2D–2F). Importantly, the PZ-4 (H1N2) vaccinated ferrets showed absolutely no sign of fever and remained in healthy condition following challenge with Sw09 (H1N2) virus.

### EDV effectively cleared influenza virus in respiratory organs

**Fig 3:**
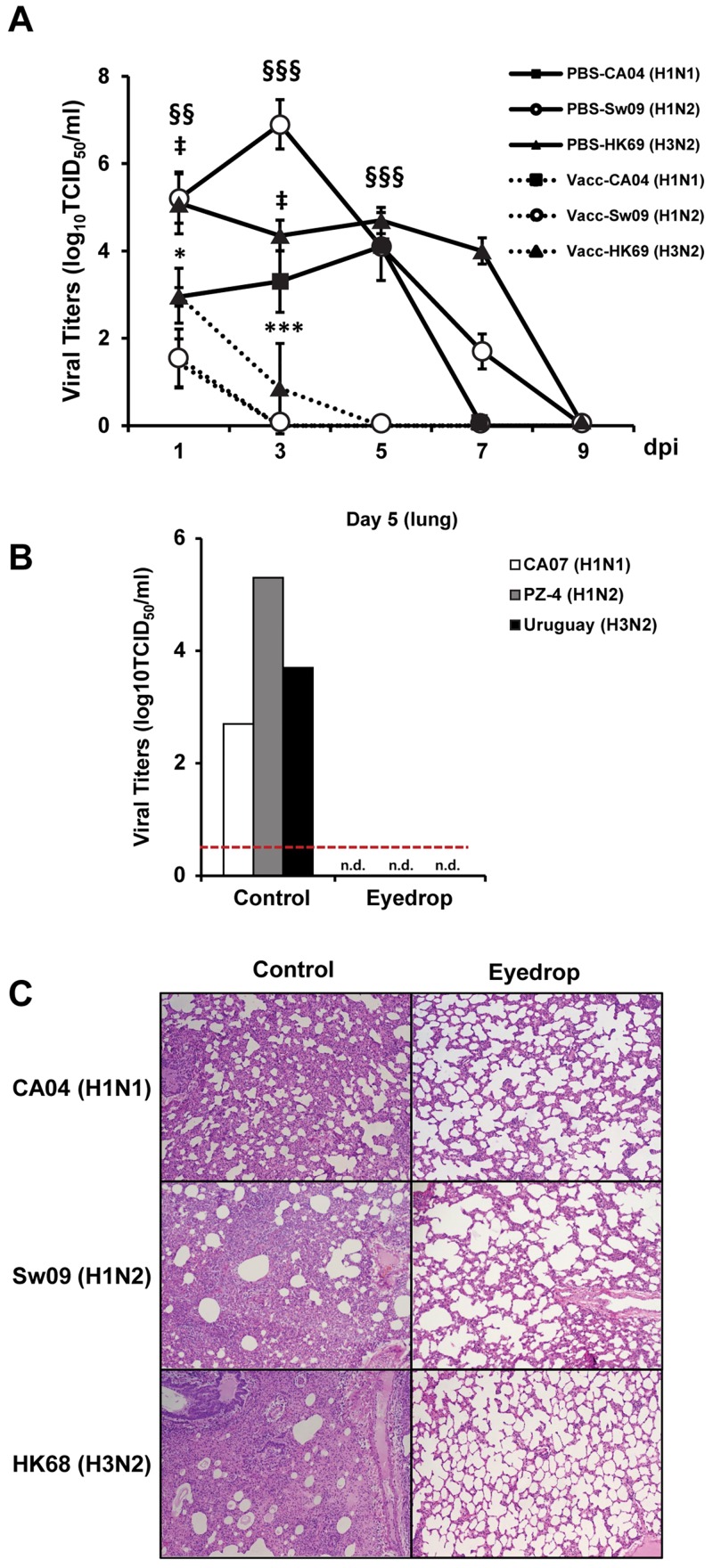
Successful viral clearance by eyedrop influenza vaccination. After intranasal challenge with CA04 (H1N1) or Sw09 (H1N2) or HK68 (H3N2), viral titers in nasal secretion samples taken at 9 dpi (A) or homogenized lung tissues at 5 dpi (B) were measured by plaque assay. Half of all mock or vaccinated ferrets in each group were sacrificed at 5 dpi for the lung tissue preparation (n = 3 for each group). (C) Hematoxylin- and eosin-stained sections of lung tissue collected from: CA04 (H1N1; Top panels) or Sw09 (H1N2; Middle panels) or HK68 (H3N2; Bottom panels) challenged ferrets. Left panels, PBS administered ferrets (HI titer <20); right panels, eyedrop vaccinated ferrets (HI titer ≤40). Magnification 200×. *p < 0.05, ***p < 0.001 compared with the findings in the CA07 (H1N1) LAIV-vaccinated and CA04 (H1N1) challenged group; §§ p < 0.01, §§§ p < 0.001 compared with findings in the PZ-4 (H1N2) LAIV-vaccinated and Sw09 (H1N2) challenged group; ‡p < 0.05 compared with the findings in the Uruguay (H3N2) LAIV-vaccinated and HK69 (H3N2) challenged group. i.n., intranasal, n.d., non-detected.

To assess the presence of live virus in the nasal passages of challenged ferrets, influenza virus titers were measured in nasal lavage samples at designated time points post-infection. Proliferating viruses were detectable until 7 dpi with maximum titers of 3.0–4.0 TCID50/ml for CA04 (H1N1), 6.5–7.2 TCID50/ml for Sw09 (H1N2), and 4.7–5.5 TCID50/ml for HK69 (H3N2) in mock-immunized animals (Fig 3A). In contrast, viral propagation in EDV counterparts was significantly abrogated beginning at 3 dpi in CA04 (H1N1) and Sw09 (H1N2) challenged groups and from 5 dpi in the HK69 (H3N2) group (Fig 3A). Furthermore, in order to confirm viral clearance in the lower respiratory organs, we also assessed the viral titers present in homogenized total lung tissues of control and vaccinated ferrets at 5 dpi. As shown in the Fig 3B, viruses were not detected in homogenized lung tissues of immunized animals, but present at levels demonstrative of a persistent infection in control animals.

Histopathological comparisons of lung tissues prepared from immunized ferrets at 5 dpi demonstrated the presence of protective immunity in EDV ferrets. Challenge with all infectious viral strains resulted in extensive immune cell infiltration into the perivascular space and alveoli, accompanied by a marked reduction in the number of alveoli in unimmunized ferrets; whereas the lung tissue of eyedrop-vaccinated ferrets was nearly intact with regular alveolar morphology and no interstitial pneumonia (Fig 3C and S2 Fig). Overall, these results demonstrate that LAIV eyedrop inoculation yields a sufficient immunological response to confer protective immunity against infectious respiratory challenge with either homologous or heterologous influenza infection in ferrets.

### Influenza EDV are safe in ferret eyes

**Fig 4:**
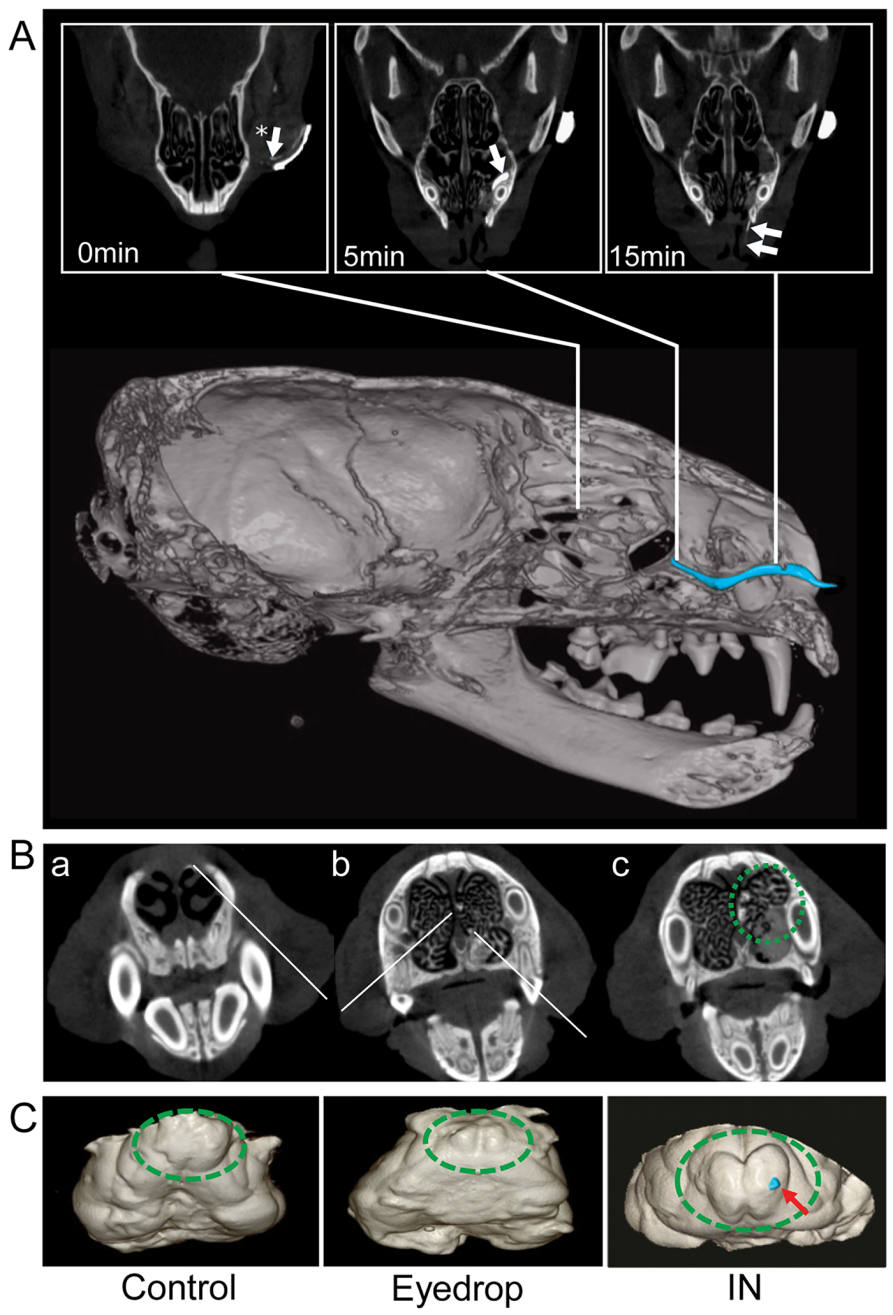
CT images of head and brains of ferrets after PBS, eyedrop, or intranasal (i.n.) treatment of a contrast medium. (A) Three-dimensional (3D) reconstruction images of the ferret head 30 min after inoculation of eyedrop iodine contrast agent (blue line); upper panels, transverse section images of head CT of ferrets 0, 5 and 15 min after inoculation of eyedrop iodine contrast agent. White lines indicate the position of contrast medium when each transverse section images was taken. (B) Coronal images of brain CT of ferrets enhanced at 30 min after i.n. iodine treatment at the level of the premaxilla (B-a), the maxilla (B-b) and the palatine (B-c). White lines indicate the white spots of contrast medium; a green dashed-circle indicates the area that white contrast medium was scattered. (C) 3D reconstruction images of the brains, which were removed from ferrets after 30 min PBS (a) or eyedrop (b) or i.n. (c) treatment of a contrast medium. Green dashed-circle, olfactory bulb; blue spot and red arrow, contrast medium.

To exclude the possibility of CNS contamination via the olfactory bulbs, facial CT scans were taken 30 minutes after administration of contrast medium by eyedrop or intranasal spray methods. When given ocularly, the contrast medium was not absorbed by the eye, but rather ran through the tear duct and extended from the lower punctum to the nasal cavity via the nasolacrimal duct (NLD) (Fig 4A). In comparison, the intranasal administration of contrast medium followed the same method used for the vaccination of the FluMist^®^, which disseminates the vaccine components with high pressure to the nasal mucosa (Fig 4B). As shown in Fig 4C, the ipsilateral olfactory bulbs of the intranasal treatment group was contaminated by the contrast medium (blue dot in dashed circle), which was not present in EDV ferret.

**Fig 5:**
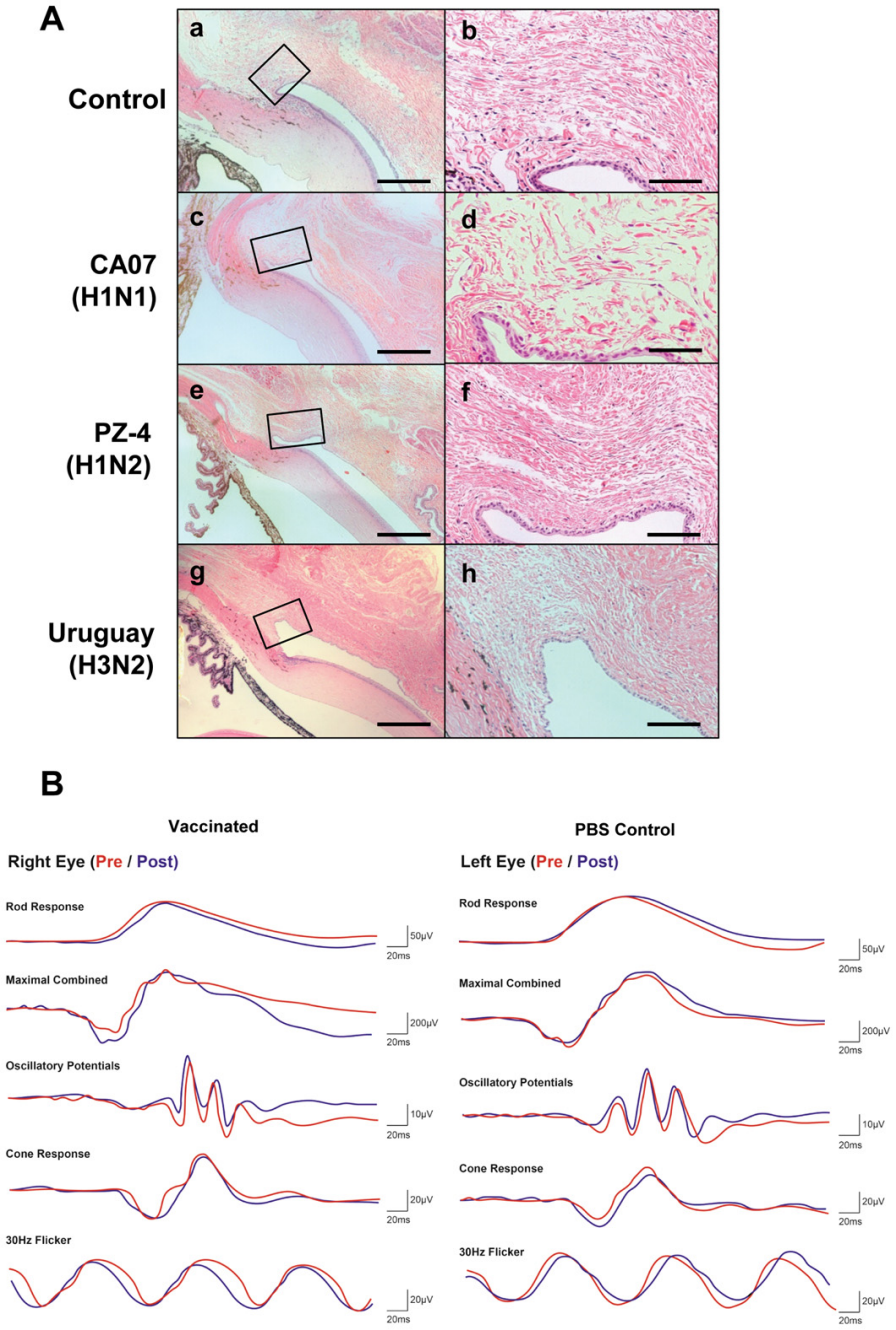
Eyedrop inoculation of vaccine-strain LAIVs does not induce inflammation in ferret eye tissues. (A) Histology of eye tissues 24 h after PBS (a, b) and various LAIVs including CA07 (H1N1) (c, d), PZ-4 (H1N2) (e, f), Uruguay (H3N2) (g, h) on eyes of ferrets. b, d, f, and h are magnified ones of the rectangle area in their paired pictures. (B) After the ferrets were dark-adapted for 12 h, full-field ERG recording was performed on both eyes of under the anesthesia (Pre), then retaining the ferrets in dark room for ferret’s dark-adaptation eyedrop CA07 (H1N1) LAIV was administered only on right eye. After 24 h the eyedrop vaccination, full-field ERG recording was performed again on both eyes of under the anesthesia (Post).

We next examined whether vaccinated LAIV materials were able to elicit acute eye inflammation. Eyes and the surrounding tissue were excised 24 h after LAIV infection and stained with H&E. As shown in Fig 5A, conjunctiva tissues displayed normal histopathology in all eyes treated with LAIVs, with additional mononuclear cell infiltration in superior fornix and bulbar conjunctiva and normal number of goblet cells in tarsal conjunctiva areas. Electroretinogram (ERG) was also used to evaluate the effect of vaccination on ocular function. ERG amplitudes remained consistent with no significant changes in A- and B-waves measured in scotopic or photopic conditions when comparing readings obtained before and one day after EDV. Within individual comparisons, no observable differences were found between the vaccinated right eye and non-treated left eye (Fig 5B).

Additionally, to check indirectly the possibility of proliferation and the virulence of vaccinated virus, we picked up PZ-4 (H1N2) vaccine strain virus, since its counterpart-challenge strain showed most severe virulency, and measured the body temperature change after vaccination of PZ-4 (H1N2) (S3 Fig). For seven days after the vaccination, body temperatures of PZ-4 vaccinated ferrets were remained within the range of normal body temperature while body temperatures of Sw09 (H1N2) challenged ferrets were spiked up to 41°C (PBS group in Fig 2E). Moreover, virus tittering was checked in eye mucosa. However, there was no replication in the tissue (S4 Fig). Therefore, these results support that the vaccinated LAIV does not proliferate in the administered tissues.

Taken together, these data demonstrates that EDV with live vaccine-strain influenza virus is anatomically, histologically, and functionally safe in ferrets, with no transfer of vaccine material contamination to the adjacent CNS area and nor inflammation in conjunctival area.

## Discussion

Eye mucosa is constantly exposed to the external environment except during sleep, inadvertently serving as a port of entry for numerous foreign antigens; thus, it has developed several different defense mechanisms. Mucosa-associated lymphoid tissue (MALT) is an important component of the immune system, especially that located at the conjunctiva—called the conjunctiva-associated lymphoid tissue (CALT)—which composes the craniofacial immune system along with the tear duct-associated lymphoid tissue (TALT) and the nasopharynx-associated lymphoid tissue (NALT) [26, 27]. EDV elicits responses in all of these tissue areas CALT, TALT, and NALT, evoking a strong antigen specific response and protective immunity.

In this study, we demonstrate that the eye mucosa serves as an effective route of vaccine administration against influenza virus in ferrets. We also show that EDV induces both systemic and mucosal immunities, as determined by the increases in serum and mucosal anti-LAIV HI titers (Fig 1). While previous data gathered in mouse models already established that EDV can provoke immunogenicity [13, 28]. However, this study with ferrets is significant in that we evaluated the clinically meaningful level of protection through an actual post-vaccination challenge. Mice are not natural hosts of influenza and do not demonstrate the classical changes in body weight and temperature were in response to infection. This study confirmed that influenza-like clinical signs are evident in the non-vaccinated ferrets but not in those that received EDV. Regardless of the influenza subtype (H1N1, H1H2, or H3N2), EDV were shown to be effective. In addition, an actual post-vaccination challenge with viruses clearly demonstrates that animals in the EDV group were protected from both an acute rise in viral titer and histological damage within the lung. Unfortunately, we failed to directly compare the efficacy of different subtypes of EDV between other groups. Even though the maximum infection dose of the challenge viruses were inoculated to corroborate the protective efficacy of the eyedrop vaccine, the different infection doses (10^5.0 TCID50 for CA04 and 10^6.0 TCID50 for Sw09 and HK68) could limit the comparison of protective efficacy between groups.

Although EDV are effective, there is a limitation in human application due to the potential of profound side effects. Importantly, we show that the route of eye mucosa administration was safe with respect to CNS contamination. Since the ocular route is connected to the nasal cavity through the NLD, CNS side effects have always been suspected in the application of EDV, as with intranasal vaccination. Due to the risk of Ag delivery to the CNS through the nasal cavity, there is a critical controversy regarding the safety of these vaccines. Previous experiments with ferrets conclusively demonstrated that i.n. inoculation of influenza virus invades CNS not only through olfactory bulb, but also through vestibulocochlear nerve [29]; thus, we confirmed CNS safety using more practical method by Micro CT scan. Notably, ferrets with i.n. administration of contrast medium exhibited uptake within the ipsilateral olfactory bulb, while eyedrop administration showed no sign of contrast medium infiltration.

Altogether, this study concluded that the differences in the risk of CNS side effects are the result of the several independent characteristics for each route. First, there are differences in the area of Ag dissemination when administrated intranasal spray can infiltrate into the CNS through the olfactory nerve fibers in cribriform plate or through Eustachian tube in nasopharynx [29]. Conversely, EDV flows to the nasal cavity through the NLD underneath the inferior turbinate, creating a stream on the lateral wall of nasal cavity. Considering the law of gravity and the fact that tear flow follows the base of nasal cavity, it would be very difficult for Ag to access the CNS via this route. Another characteristic that should be considered is the difference in the velocity between the two administration methods. Speed of commercial i.n. spray ranges from 6.7 to 19.2 m/s. In contrast, the average rate of tear drainage is 0.4 to 0.6 μL/minute, and the velocity of the flow to NLD increases up to 22 μL/minute when administered by eyedrop. Moreover, considering that around 90% of tears are reabsorbed into the NLD mucosa, the flow rate of eyedrop is fairly slower compared to that of i.n. spray [30, 31]. Therefore, the risk of CNS adverse effect is minimal for eyedrop administration, since it is physically unreachable. The comparison of similarities and differences are summarized in Table 2.

**Table 2:** The comparison of similarities or differences between EDV and IN vaccination.

	Eyedrop	Intranasal
**Vaccine volume**	5ul/eye (mouse)	10ul/nose (mouse)
**Immune induction**	Systemic and Mucosal (mouse)^[^^13^^]^	Systemic and Mucosal(mouse & human)^[^^2^^]^
**CNS infiltration**	No (mouse)^[^^13^^]^	Yes (mouse & human)^[^^27^^]^
**Type of sialic acid on mucosa**	α2,3-linked sialic acid (human)^[^^28^^,^^29^^]^	α2,6-linked sialic acid (human)^[^^28^^,^^29^^]^
**Velocity of vaccine administration**	Slow (0.4~0.6 uL/min; human)^[^^26^^,^^27^^]^	Fast (6.7 ~ 19.2 m/s; human)^[^^26^^,^^27^^]^

The superscripts, number of reference.

Local side effects, such as tearing or red-eye, were anticipated with direct vaccine administration to the mucosa; however, we were unable to detect any histological signs of local adverse effects, owing to virus dilution by the circulation of tears. In addition, the distribution of sialic acid in the eye mucosa is not fitting for influenza virus tropism. In the ferret, sufficient amounts of α2,3-linked sialic acid (SA 2,3) is expressed in the conjunctiva, while α2,6-linked sialic acid (SA 2,6) is mainly expressed in nasal cavity and upper respiratory tract mucosa similar to humans [32, 33]. Because influenza viruses mainly bind to SA 2,6, administering LAIV with eyedrop cannot cause conjunctival inflammation. Furthermore, our study used ERG to ascertain the effect of eyedrop vaccination on ocular function. ERG usually consist of two sequential waveforms: the first, termed the A-wave, is a negative wave, which reflects the photoreceptor function, whereas the second, or B-wave, is a positive wave originating from the main bipolar cells. Importantly, no changes reflective of retinal damage or vitreous haziness were observed in our readings, as interpreted from post-vaccination change in wave amplitude, and confirmed that eyedrop vaccination had no adverse effects on retina and vitreous.

There has been insufficient amount of studies conducted regarding the eye mucosa up to date. This study has confirmed the protective effect of EDV against influenza virus while proving the safety of this type of vaccine compare to the intranasal ones. However, further studies regarding the in-depth mechanism of immune induction in eye mucosa are necessary. Moreover, studies regarding the cross protection and application of trivalent or quadrant vaccination are in need considering the commercial use of the EDV since this study only focused on the protection against homotypic influenza virus. Lastly, the subject of the study is limited to the influenza A virus. Although influenza A virus is the major infectious influenza virus in human, a need to devise more effective influenza B virus vaccine is on the rise. Recently, our group reported that mouse adapted influenza B virus showed transmissivity in ferrets [34]. However, in the study of influenza B virus transmission between ferrets, the utility for ferret is not established. Therefore, based on the study, further evaluation to measure the effectiveness of EDV against influenza B virus is also necessary.

In conclusion, this study demonstrates that eyedrop vaccination elicits an immunological response sufficient to produce protective immunity, and poses less risk of CNS side effects. EDV is exceptionally convenient when compared to other methods of immunization. Furthermore, EDV maximize the advantages of mucosal vaccines; although the general mechanism and application for various pathogens should be assessed with further studies. Nevertheless, our data confirms the safety and efficacy of EDV, and highlights this method as a suitable and attractive candidate for the prevention of epidemics in the future.

## Supporting Information

S1 Fig Levels of anti-EDV Abs in serum or nasal wash by ELISA. (DOCX)

S2 Fig Comparison of clinical scores of H&E stained lung slides between control or EDV ferrets. (DOCX)

S3 Fig Body temperature changes after the PZ-4(H1N2) vaccination in ferrets. (DOCX)

S4 Fig No replication of EDV virus in eye tissues after inoculation in vaccinated ferrets. (DOCX)

## Abbreviations

EDV: eyedrop vaccination
CNS: central nervous system
Ag: antigen
LAIV: Live-Attenuated Influenza Vaccine
Abs: antibodies
NLD: nasolacrimal duct
ERG: electroretinogram
