# Supplementary material for: Eyedrop Vaccination Induced Systemic and Mucosal Immunity against Influenza Virus in Ferrets
Source: PLoS One. 2016 Jun 22;11(6):e0157634. doi: 10.1371/journal.pone.0157634 (PMC4917170; doi:10.1371/journal.pone.0157634)

**S1 Fig. Levels of anti-EDV Abs in serum or nasal wash by ELISA.**

After EDV inoculation of CA07 (H1N1) or PZ-4 (H1N2) or Uruguay (H3N2), anti-ferret IgG titers in serum samples of 2-week post EDV vaccinated ferrets or IgA titers in nasal wash samples of 2-week or 4-week post EDV vaccinated ferrets were measured by ELISA (n=3 for each group).

**S1 Fig.**


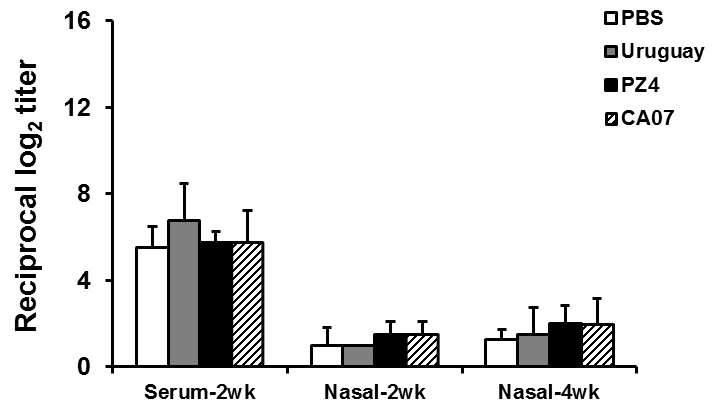

Supplement: S1 Fig — (DOCX) [file pone.0157634.s001.docx]
