# Supplementary material for: Eyedrop Vaccination Induced Systemic and Mucosal Immunity against Influenza Virus in Ferrets
Source: PLoS One. 2016 Jun 22;11(6):e0157634. doi: 10.1371/journal.pone.0157634 (PMC4917170; doi:10.1371/journal.pone.0157634)

**S2 Fig. Comparison of clinical scores of H&E stained lung slides between control or EDV ferrets.**

Semi-quantitative analysis of lung inflammation severity in influenza virus-challenged ferrets was performed with some modification as reported elsewhere [24] for the alveoli. For the severity of inflammation in the alveoli, we scored (1-2) no infiltration of inflammatory cells and intact alveoli size, (3-4) mild infiltration of inflammatory cells and mildly shrunken alveoli, and (5-6) marked infiltration of inflammatory cells and extremely shrunken or disappeared alveoli. The cumulative scores for severity and size of inflammation provided the total score per animal. Slide numbers per lung, 5 to 7 slides. **p* < 0.05 compared with the findings in the control group. Statistical analyses were conducted by the student’s *t-test*.

**S2 Fig.**


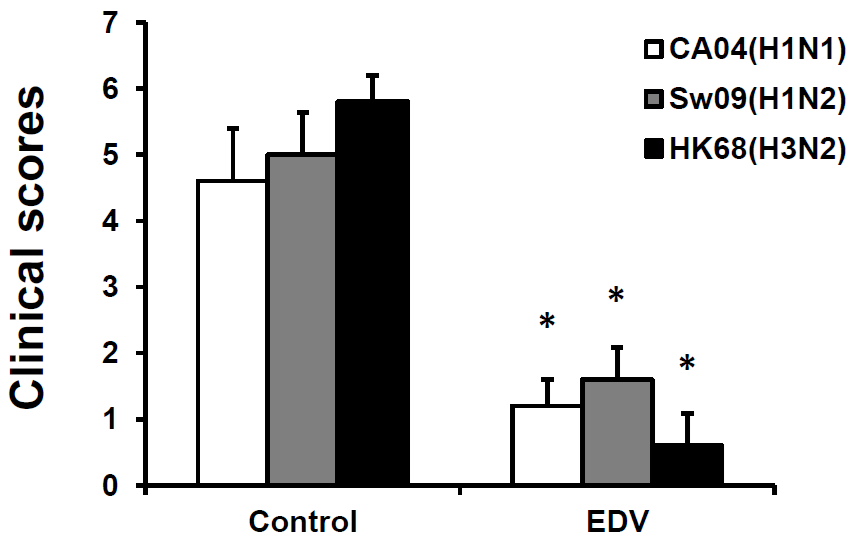

Supplement: S2 Fig — (DOCX) [file pone.0157634.s002.docx]
