# Supplementary material for: Eyedrop Vaccination Induced Systemic and Mucosal Immunity against Influenza Virus in Ferrets
Source: PLoS One. 2016 Jun 22;11(6):e0157634. doi: 10.1371/journal.pone.0157634 (PMC4917170; doi:10.1371/journal.pone.0157634)

**S3 Fig. Body temperature changes after the PZ-4(H1N2) vaccination in ferrets.**

15- to 16-week-old ferrets were purchased from Marshall BioResources (North Rose, NY, USA). All animals were confirmed seronegative for vaccine strains of PZ-4 (H1N2) influenza A viruses used in this study by serologic assay. For conjunctival immunizations, three ferrets were anesthetized by inhalation of isoflurane, and then 10^5^.0 TCID_50_ of the PZ-4 (H1N2) live attenuated influenza vaccines (LAIVs) which are resolved 100 µL PBS were dropped to each eye (200μL/head). After the vaccination, body temperatures were monitored for seven days. Eyedrop, Sw09 (H1N2) challenged ferrets in PZ-4 (H1N2) eyedrop vaccinated group in figure 2E; PBS, Sw09 (H1N2) challenged ferrets in PBS treated group in figure 2E. Dotted lines, normal body temperature range. **p < 0.01, ***p < 0.001 compared with the findings in the PBS control group (n=3 for each group). Statistical analyses were conducted by the student’s *t-test*.

**S3 Fig.**


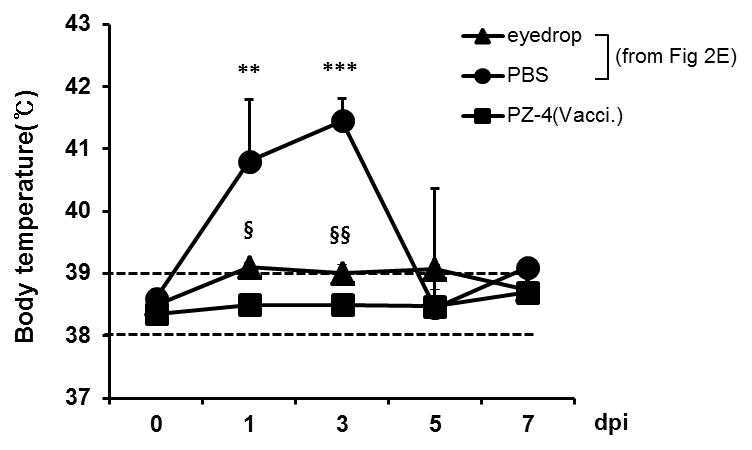

Supplement: S3 Fig — (DOCX) [file pone.0157634.s003.docx]
