# Supplementary material for: Eyedrop Vaccination Induced Systemic and Mucosal Immunity against Influenza Virus in Ferrets
Source: PLoS One. 2016 Jun 22;11(6):e0157634. doi: 10.1371/journal.pone.0157634 (PMC4917170; doi:10.1371/journal.pone.0157634)

**S4 Fig. No replication of EDV virus in eye tissues after inoculation in vaccinated ferrets.**

After EDV inoculation of CA07 (H1N1) or PZ-4 (H1N2) or Uruguay (H3N2), viral titers in eye tissue samples taken at 24 hour post infection were measured by plaque assay (n=3 for each group). Dotted line indicates the virus detection limit (1.7 log_10_TCID_50_/ml).

**S4 Fig.**


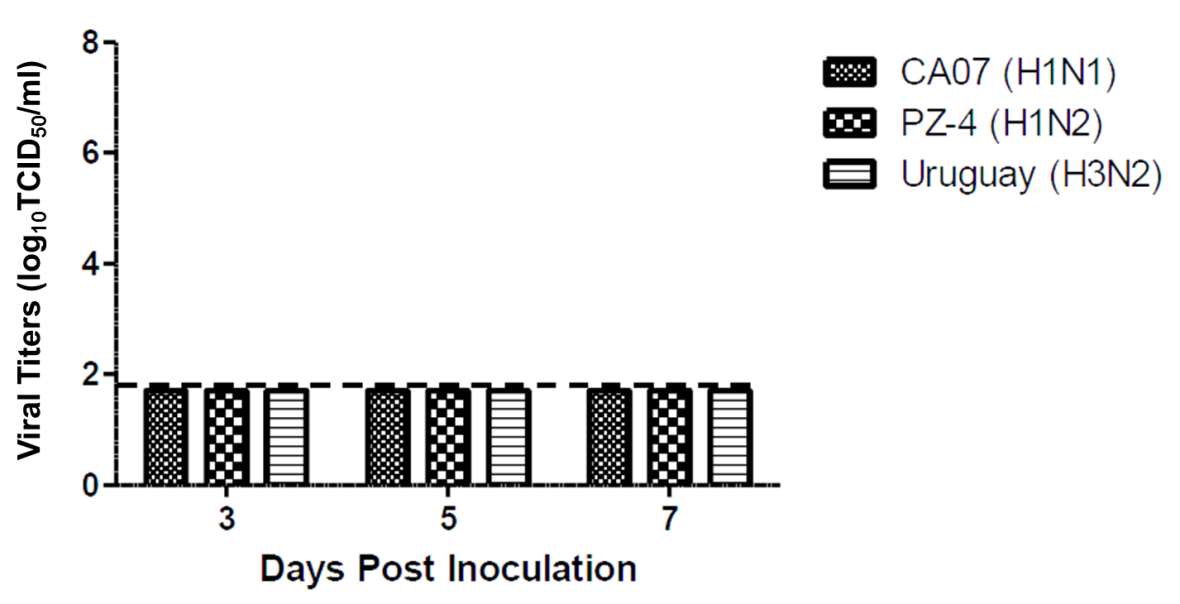

Supplement: S4 Fig — (DOCX) [file pone.0157634.s004.docx]
